# Application of digital twins for simulation based tailoring of laser induced graphene

**DOI:** 10.1038/s41598-024-61237-6

**Published:** 2024-05-06

**Authors:** José Carlos Santos-Ceballos, Foad Salehnia, Alfonso Romero, Xavier Vilanova

**Affiliations:** Universitat Rovira i Virgili, Microsystems and Nanotechnologies for Chemical Analysis (MINOS), Tarragona, Spain

**Keywords:** Materials for devices, Graphene, Design, synthesis and processing

## Abstract

In the era of man–machine interfaces, digital twins stand as a key technology, offering virtual representations of real-world objects, processes, and systems through computational models. They enable novel ways of interacting with, comprehending, and manipulating real-world entities within a virtual realm. The real implementation of graphene-based sensors and electronic devices remains challenging due to the integration complexities of high-quality graphene materials with existing manufacturing processes. To address this, scalable techniques for the in-situ fabrication of graphene-like materials are essential. One promising method involves using a CO_2_ laser to convert polyimide into graphene. Optimizing this graphitization process is hindered by complex parameter interactions and nonlinear terms. This article explores how these digital replicas can enhance the fabrication of laser-induced graphene (LIG) through laser simulation and machine learning methods to enable rapid single-step LIG patterning. This approach aims to create a universal simulation for all CO_2_ lasers, calculating optical energy flux and utilizing machine learning to control and predict LIG conductivity (ability to conduct current), morphology, and electrical resistance. The proposed procedure, integrating digital twins in the LIG production process, will avoid or reduce the preliminary tests required to determine the proper laser parameters to reach the desired LIG characteristics. Accordingly, this approach will reduce the time and costs associated with these tests and thus increase the efficiency and optimize the procedure.

## Introduction

In today's landscape of human–machine interfaces, digital twins are emerging as a powerful technology for supporting innovation and optimization. They provide virtual representations of physical machines, complicated processes, and complex systems through computational models. Digital twins are increasingly used in material science and chemical synthesis to accelerate the development of new materials innovation. In material science, digital twins can depict the progression of materials structure, processes, and performance^1^. They can also be used to predict the behavior of materials under different conditions, such as high temperature or pressure^2^. Digital twins can optimize the manufacturing process and ensure that the materials meet the required specifications. Our article explores the utilization of these digital replicas to enhance the fabrication process for LIG.

Graphene is a highly versatile two-dimensional (2D) carbon nanomaterial known for its outstanding mechanical, electrical, thermal, and optical properties^3^. In 2004, the stability and high electron mobility of this atom-thin material, consisting of a hexagonal carbon lattice, were initially documented through the mechanical exfoliation of natural graphite^4^. Several methods have been proposed to develop graphene-like materials with methods suited to scalable fabrication. These include chemical vapor-deposited graphene, reduced graphene oxide, and liquid-phase exfoliated graphene^5^. Each approach can be assessed in terms of graphene quality and throughput^6^. Nevertheless, these conventional approaches are often burdened by disadvantages such as intricate manufacturing procedures, rigorous preparation conditions, and limited control over the resulting shape^7^. While patents have existed for producing graphitic materials through laser graphitization since 1972, there was limited understanding of the materials produced. Tour and his colleagues took the lead in developing a method for directly creating porous three-dimensional (3D) LIG using laser graphitization of polyimide with CO_2_ lasers, marking a significant advancement^8,9^. The unique attributes of LIG, including high porosity, mechanical flexibility, and exceptional electrical conductivity, position it as an ideal material for various applications, from microfluidic systems and electronic devices to catalysis systems, water purification, and sensors^7,8,10–12^. The utilization of laser graphitization technology presents an exciting opportunity for directly integrating porous 3D graphene electrodes onto polymer substrates. While polyimide stands out as the most promising material for this purpose, researchers such as Tour and others have successfully demonstrated the generation of graphene-like carbon using various synthetic polymers and natural materials such as wood, food, textiles, charcoal, and anthracite coal^13^. This process involves multiple laser passes at lower fluences, sometimes employing defocused lasers to enhance the overlap of laser paths, thereby achieving high-quality LIG.

The optimization of the laser graphitization process requires the identification of appropriate process parameters, such as average laser power and scan speed, tailored to each specific type of material^14^. These parameters enable the direct writing of 3D porous structures with graphene-like carbon. Avoiding nonoptimal laser parameters that can lead to undesired effects, such as material ablation, redeposition, oxidation, or incomplete graphitization, is crucial. Rigorous and efficient optimization strategies are needed to address this complex material challenge^15^. Today, computational methods play a crucial role as a complement to experimental approaches in the exploration, design, and optimization of materials. Computational techniques offer the capability of finely tuning simulation conditions, such as the adjustment of a single parameter or a set of parameters^16^. Recently, machine learning (ML)-based tools have been shown to be alternative approaches to accelerate the discovery and design of materials^17^. An artificial neural network (ANN) is an ML algorithm inspired by biological neural networks^18^. ANNs are commonly used for classification tasks. They are composed of connected perceptron (artificial neuron), organized in layers, which are divided into input (features data), hidden (where computation occurs) and output (class labels) layers. To classify data, ANNs use a labeled dataset, which means that each data point has a known label, the labels are added to the dataset manually. During training, backpropagation is employed to iteratively adjust the weights and biases of the network. In this way, ANNs learn to make predictions on new data, by correlating the labels with the input features^19,20^. In various research, ANNs have been employed to predict the properties of materials^21–23^. Gaussian process regression (GPR) is another supervised learning method used in this field^24^, offering a nonparametric Bayesian approach toward regression to model complex relationships between inputs and outputs^25^. In other words, it is possible to obtain models that relate different independent variables and a dependent variable or output using this algorithm.

The main objective of this article is to develop a laser simulation model combined with a machine-learning technique that can be used to facilitate the tuning of laser parameters to produce LIG with the re characteristics. This would significantly reduce the time and cost associated with LIG production optimization, preparing a method for scalable, efficient, and cost-effective LIG production. LIG is a promising material for the fabrication of graphene-based electronic devices. However, the production of LIG can be challenging and time-consuming, as it requires careful optimization of the laser parameters. The simulation model developed in this study will be able to predict the characteristics of LIG based on the lasing parameters. This model can then be used to automate the tuning of laser parameters to produce LIG with the desired characteristics, according to the application. The machine learning technique developed in this study will be able to predict the electrical resistance, morphology, and conductivity of a fabricated LIG layer. Using this method will optimize the LIG production process further and ensure that the desired characteristics are achieved. The successful development of this digital twin will have a significant impact on the field of graphene-based electronics.

## Methods

### Experimental setup and methods

The LIG was fabricated using a commercial polyimide film (50 µm thickness) as the substrate. Initially, the substrate was cleaned with acetone and then attached to a polyethylene sheet using double-sided tape. LIG was synthesized using a CO_2_ laser system (SYNRARD 48-2) with a wavelength of 10.6 μm and a maximum power of 25 W. The laser system features a field size of 27 × 27 mm, with a precise spot size of 1/e^2^ at 116 µm and a working distance of 80 mm. Its typical depth of field is ± 0.4 mm, and it operates with a maximum incident angle of 5 degrees. The system achieves remarkable efficiency, allowing for a maximum marking speed of 5000 mm/s and a frequency range of 0 Hz to 20 kHz. The laser was focused onto the polyimide surface, and scanning of the laser beam over the surface was fixed at different duty cycles, speeds, and frequencies (parameters adjusted for the used laser). This precise scanning process allowed for the selective conversion of the polyimide film into graphene. A Scios 2 DualBeam field emission scanning electron microscope (FESEM) was used to analyze the morphology. The Raman analysis was conducted using a Raman spectrometer (Renishaw plc) with a coupled confocal Leica DM2500 microscope (Leica Microsystems GmbH) with a laser of 514 nm wavelength.

### Simulation of laser direct writing

One of the characteristics that has been reported in the literature that most influences LIG obtaining is laser fluence (LF), which is the amount of energy delivered in a unit area (J/mm^2^). To obtain this value (and other interesting parameters, such as power density or peak power), a MATLAB simulation code was implemented to model the laser writing process for LIG fabrication. The configurable parameters of the laser were used as input values, and beam diameter, maximum power, and pulse rise time were used as fixed specifications of the laser used in this work. The entire writing process is simulated using a Simulink model to obtain other relevant parameters to characterize the writing process as total energy delivered to the substrate, fluency values, power density, and peak power for various combinations of the initial parameters to be fixed in the laser system: laser frequency, duty cycle, and speed. Additionally, the impact of overlapping laser pulses on the writing process is assessed, and the energy deposition is calculated when the period of each pulse is less than the rise and fall time of the laser, especially at high frequencies. This methodology allows for the exploration of a broad range of conditions while maintaining a foundation in practicality, ensuring the comprehensive analysis and fine-tuning of LIG fabrication.

**Figure 1:**
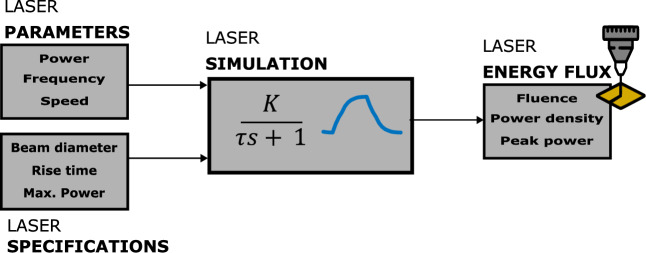
Block diagram of the laser direct writing simulation.

**Figure 2:**
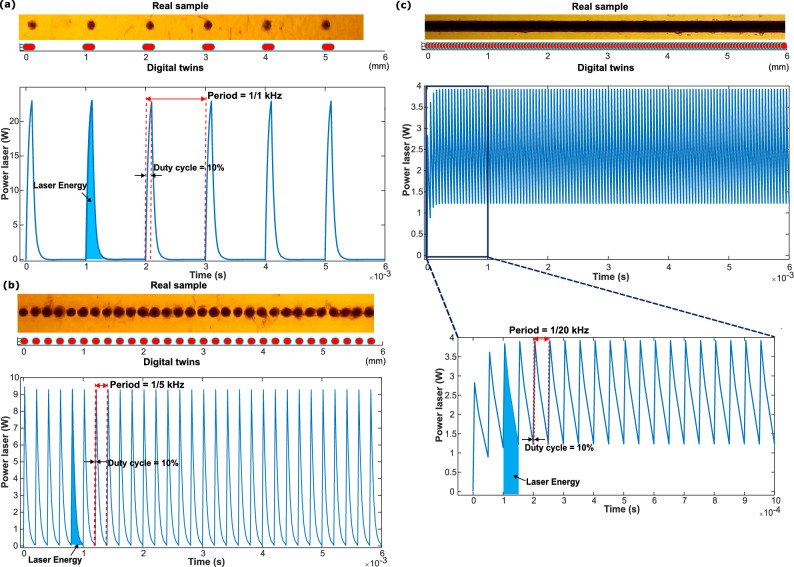
Image of the real samples, simulation of the laser drawing, and diagram of simulation of the laser power with different laser parameters: duty cycle 10%, speed 1000 mm/s, frequency (**a**) 1 kHz, (**b**) 5 kHz and (**c**) 20 kHz.

The laser direct writing was simulated over time while drawing a straight line 6 mm long and a width equal to the beam diameter (116 µm), using MATLAB Simulink as a simulation tool. Considering that a pulsed laser is used, the more appropriate model is a first-order system without delay, with the following transfer function, f(s) = K/(τs + 1), where K is the maximum power of the laser (25 W) and τ is the rise time (40 µs). As an input signal, a pulse width modulation (PWM) is used; its parameters match the laser’s frequency and power duty cycle. Figure 1 shows a block diagram of the laser direct writing simulation, and Fig. 2 shows the results of the simulation for a set of parameters.

For the laser fluency calculation, we use the formula LF = E/A, where E is the energy delivered by the laser and A is the area on which the laser is incident. For the energy calculation, the area under the curve was obtained from the graph of power vs time resulting from the simulation. The area of laser exposure was measured according to the parameters of the laser, which accurately simulated the path of the laser beam in off and on times. Additionally, the determination of peak power and power density is essential for a comprehensive analysis of the process. Peak power is calculated by examining the highest point on the power vs. time graph, corresponding to the laser pulse's maximum energy release. This value, designated 'K' in our first-order laser model, represents the laser's maximum instantaneous power output. The power density, on the other hand, is the spatial distribution of this power within the beam area. It is determined by dividing the peak power by the area of the laser beam, considering the beam diameter as a fixed specification and the laser scanning speed as a variable specification. This parameter helps us understand how concentrated the energy delivery is within the beam, aiding in optimizing the laser writing process for precise and controlled LIG fabrication. Together with laser fluency, these calculations contribute to a thorough assessment of the laser's performance and its impact on the resulting material properties. Figure 2 shows the result of the graphical simulation. The simulation also calculates and visualizes the overlapping of laser shouts and the number of pulses in the area of one beam diameter unit to calculate overlapping A more detailed exposition of this simulation methodology can be found in the supplementary information. A more detailed exposition of this simulation methodology can be found in the Supplementary information.

To verify this simulation, 648 straight lines (6 × 0.6 mm) were manufactured with different combinations of laser parameters. For this study, 6 levels of laser power (5%, 10%, 20%, 30%, 40%, and 50% of the total laser power) were used, along 12 laser speeds (50 mm/s, 75 mm/s, and from 100 to 1000 mm/s, with increments of 100 mm/s), and 9 laser frequencies (1000 Hz and from 2500 Hz to 20,000 Hz, with increments of 2500 Hz) resulting in a total of 648 possible sample combinations. For the selection of the laser parameters ranges, it was considered that laser power values above 50% would destroy the polyimide film. Moreover, energies insufficient for marking the substrate are observed at laser speeds exceeding 1000 mm/s, while speeds below 50 mm/s may lead to sample destruction.

## Experimental results and discussion

### Conductivity classification

A multilayer perceptron neural network classifier has been trained to predict whether the laser parameters are adequate to draw a continuous and thus conductive line. This is determined by the connectivity of the LIG network. A conductive LIG network is one in which the qualified LIG sheets are interconnected, forming a continuous path for electrons to flow (Supplementary Fig. S1a). Nonconductive LIG networks are those in which the LIG patterns are not interconnected (Supplementary Fig. S1b), in which the LIG sheets are disconnected by overburning (Supplementary Fig. S1c), or in which the polymer does not receive enough energy to convert to LIG (Supplementary Fig. S1d). First, the manufactured samples were classified into conductive and not conductive by measuring the electrical resistance of each sample. The electrical resistance was measured between the two ends of the line drawn by the laser, using a multimeter, this process was repeated three times to ensure accuracy and minimize potential measurement errors. The samples are considered not conductive if the measured value is above 500 kΩ. The selection of this threshold is because, during the Raman spectrum analysis of the samples with electrical resistance above this value, the formation of LIG structures was not detected, which leads us to conclude that these are not relevant to our research. Furthermore, the resistance measurement can be prone to greater variability and potential errors as the values increase. Specifically, we found that resistance measurements above 500 kΩ exhibit a pronounced increase in error, further justifying our use of this threshold. The dataset used for training is made up of the laser parameters, the simulation results, and the manual classification of each of the manufactured samples. The MATLAB Statistics and Machine Learning Toolbox have been used to process the data, as well as to train and validate the models.

**Figure 3:**
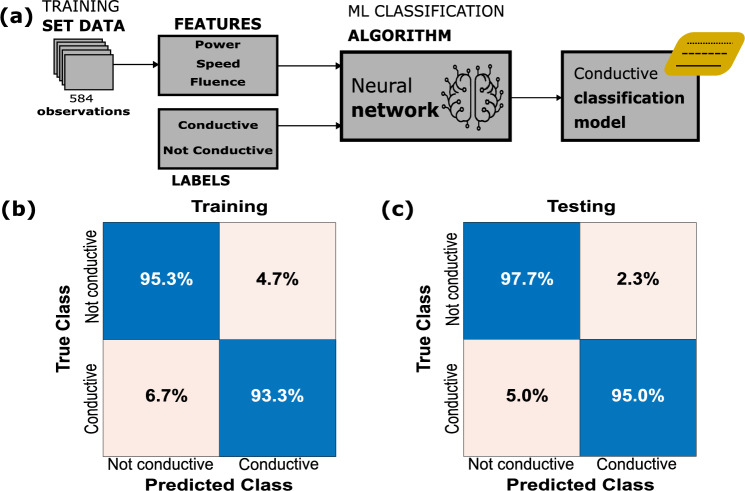
Schematic illustration shows (**a**) block diagram of conductivity classification and confusion matrix of (**b**) training and (**c**) testing procedures.

Different configurations were iterated to define the architecture of the ANN, varying the number of layers (1, 2, 3) and neurons per layer (25, 50, 75, 100), along with the selection of features, until reaching an accuracy above 90%. The neural network has 1 fully connected layer, with 100 neurons. The selected training is described in Fig. 3a. The laser's speed, power and fluency were the selected features for each observation, the output labels were conductive or not conductive. Once the hyperparameters of the neural network (number of layers, number of neurons) were fixed, were varied the distribution of the 648 observations used for training and validation of the model. Supplementary Table S1 shows that the best results were obtained for the dataset with 584 observations (90%) utilized for training the model and the remaining 64 observations (10%) reserved for evaluating model performance after fine-tuning and training. It is important to note that these observations were randomly selected for these purposes. The accuracy obtained during training (using only the samples of the training set) is approximately 94.7%, while during the test with the observations reserved for this purpose and not used in the training, the accuracy is 96.9%. The corresponding confusion matrixes are shown in Fig. 3b,c. Although a lower percentage of data for validation could be model overfitting, to avoid this phenomenon during training, a fivefold cross-validation model was used. To determine the accuracy of the models, divide the number of correct predictions by the total number of predictions and multiply by 100.

Upon analyzing the parameters chosen for the training of the neural network, it can be inferred from Supplementary Fig. S2a that the laser power duty cycle values appropriate to obtain a conductive LIG network lie between 10 and 50%. Furthermore, it can be concluded that by using a value of 5%, most of the samples turn out to be non-conductive. In Supplementary Fig. S2b, it is apparent that laser speeds within the range of 200–600 mm/s are conducive to the formation of an LIG network, with most samples outside this range being nonconductive. Supplementary Figure S2c demonstrates that the incident laser fluency on the substrate should not exceed 26 J/mm^2^ to obtain a conductive LIG network.

### Morphology classification

To investigate the correlation between the morphologies resulting from different laser fluxes and the molecular structure of LIG, Raman spectroscopy was performed along the lased lines of the conductive samples. Baseline correction was performed using asymmetrically reweighted penalized least squares (arPLS) baseline removal^26^. This method was reported by Baek et al., and it is a penalized least squares method that iteratively reweights the data points based on their deviation from a fitted baseline. The weights are asymmetric, meaning that points below the baseline are given more weight than points above the baseline. This helps to ensure that the baseline is accurately estimated, even in the presence of noise. Peak fitting was performed using MATLAB's built-in nonlinear least-squares solver. Each Raman mode was modeled as a baseline Gaussian curve. The baseline was estimated using the arPLS method described above. The nonlinear least-squares solver was used to fit the Gaussian curve to the data, minimizing the sum of the squared residuals between the data and the model. The codes used for baseline correction and peak fitting are available in GitHub-link. Abdul Hafez et al. conducted research to identify distinct thresholds corresponding to morphological transitions, classifying them as woolly fiber (WFs), cellular networks (CN), or porous formations (PF)^27^. The results of Abdul Hafez's research show a good alignment with these research findings and models.

At low laser fluxes, the Raman spectra exhibited characteristic peaks attributed to polyimide, specifically at 1395, 1601, and 1786 cm^−1^, corresponding to the C–N–C axial vibration, imide ring vibration, and C=O asymmetric ring, respectively. This suggested the initial stages of carbonization, wherein polyimide is transforming into graphene-like structures.

As the average fluency increased, the Raman peaks associated with polyimide vanished, giving rise to the appearance of G and D bands, indicative of graphitic porous LIG formation. The observed spectra displayed G and D peaks with a large full width at half-maximum (FWHM), encompassing a broadband band around the 2D peak, indicating the presence of graphitic porous structures. Notably, the spectra maintained consistent shapes along the lasing line, revealing the uniformity of the carbonization process.

**Figure 4:**
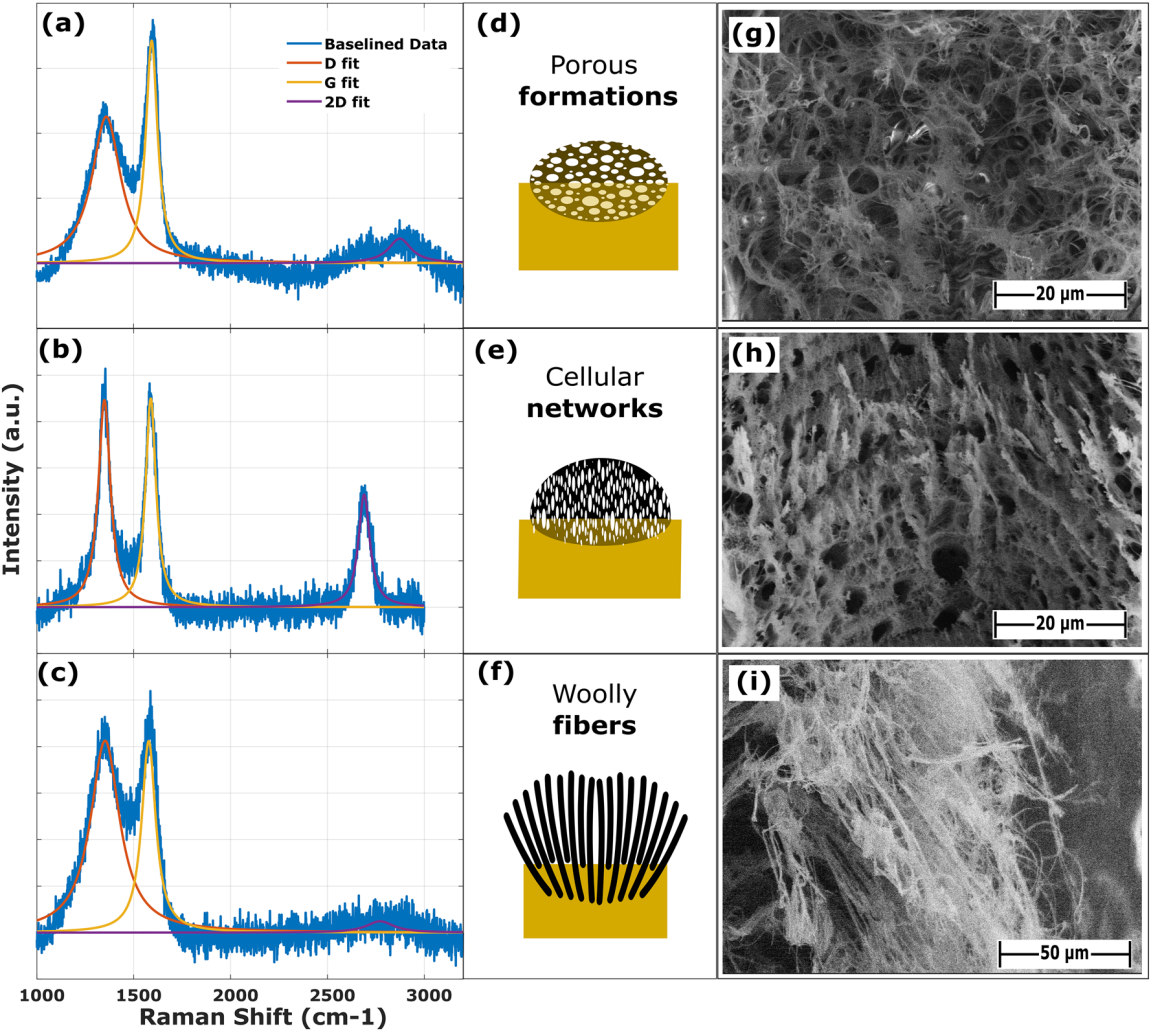
Raman spectra for three different morphologies (**a**–**c**), a schematic of morphologies (**d**–**f**) and FESEM images (**g**–**i**).

Upon further elevation of the laser flux, a sharper and well-defined 2D band materialized, accompanied by a significant reduction in the fwhm of the G and D peaks (e.g., from 150 cm^−1^ to approximately 75 cm^−1^ for the G peak, as illustrated in Fig. 4). This reduction in fwhm of the G peak was indicative of the formation of larger sp^2^ grains, coinciding with the development of well-defined graphene domains and the transformation from porous formation morphology to interconnected cellular networks of 3D graphene.

In contrast, a remarkable transition to a woolly fibrous morphology was observed at high laser fluxes, which was not evident at lower flux levels. Intriguingly, the voluminous woolly fibers displayed Raman spectra akin to those of the porous formations, with an absence of a sharp 2D peak and a larger fwhm for the G peak compared to the cellular networks. These results collectively suggested that woolly fibrous structures generally possess lower-quality sp^2^ carbon than cellular networked structures. Figure 4 shows Raman spectra for three different LIG morphologies.

In the next step, a neural network classifier was trained to predict the relationship between laser flux and LIG morphology. Considering the parameters (amplitude, position, ratio) of the Raman spectrum peaks (D, G, and 2D), which are characteristic of LIG, and by visual inspection with a microscope, the samples were classified according to their visual morphology, following the criteria defined by Abdul Hafez et al. The dataset used for training is made up of the laser parameters, the simulation results, and the morphology classification of each of the samples.

**Figure 5:**
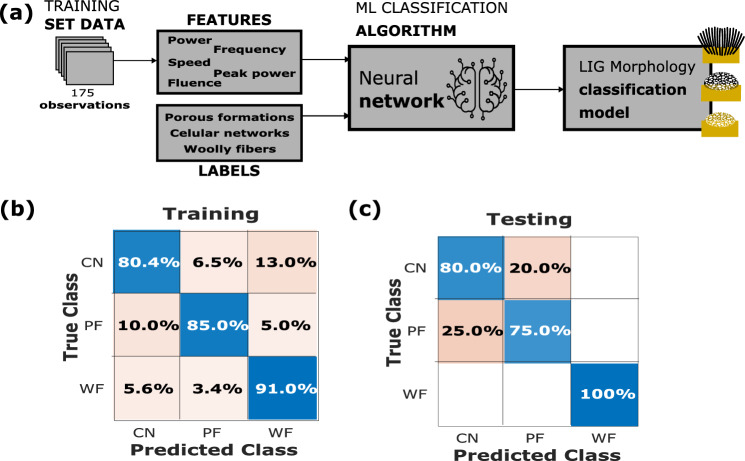
The schematic illustration shows (**a**) a block diagram of morphology classification and a confusion matrix of (**b**) training and (**c**) testing procedures.

The hyperparameters of the neural network were varied, as well as the selected features, until a model with an accuracy above 86% was obtained. The dataset has 195 samples, previously classified as conductive, in which the formation of structure LIG is present. Among these samples, 175 (90%) were employed for training the model, while the remaining 20 (10%) were reserved for testing the model, with the speed, power, and fluency of the laser, the features used, and the output labels being WF, CN or PF. The neural network has 1 fully connected layer with 100 neurons. To avoid overfitting, a fivefold cross-validation was used. Supplementary Table S2 shows the accuracy obtained during training is approximately 86.79%, and that during testing is 88.9%. Figure 5b,c) includes the confusion matrixes obtained. The implications of these findings are profound for the tailoring of LIG patterns and the optimization of their properties, particularly for applications involving electrical conductivity and morphology-dependent properties.

The relationship between the chosen features and the morphology of the LIG is reviewed (Supplementary Fig. S3a,b), and it can be concluded that to obtain LIG samples with CN morphology, the laser must be configured with low power (5–10%) and speed (50–200 mm/s) values. In the case of the WF morphology, the parameters can be set between 30–50% and 200–600 mm/s. Furthermore, to obtain LIG samples with PF morphology, high speeds (600–1000 mm/s) and power values between 20 and 40% must be used. Supplementary Figure S3c shows that there is no relationship between the fluence of the laser and the morphology of the LIG. It is worth noting that most of the samples that present PF morphology are found below 8 J/mm^2^.

### Estimation of LIG sheet resistance

**Figure 6:**
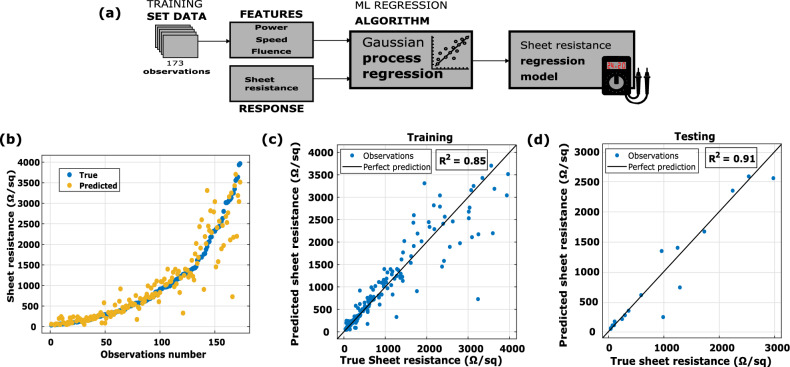
Schematic illustration shows (**a**) a block diagram of the estimation sheet resistance of LIG, (**b**) scatter plot and correlation plots of the (**c**) training and (**d**) testing procedures.

To understand how the laser flux affects the LIG resistance, a Gaussian process (GP) regression algorithm has been used to predict the value of the LIG sheet resistance, depending on the parameters with which the laser is configured. The sheet resistance (Ω/sq) is the resistance between two parallel edges of a square sheet of conductive layer material^28^. To calculate the sheet resistance of the samples that were classified as conductive, the equation R_sheet_ = R_LIG_ W/L was used, where R_LIG_ is the resistance of the measured LIG line, W is the width of the line (0.116 mm) and L is the length (6 mm). The dataset used for training consists of the laser parameters, the simulation results, and the calculated value of the sheet resistance of the samples. In this analysis, 173 samples were used, with cutoff sheet resistances below 4 kΩ/sq among the previously categorized samples as conductive, of which 156 samples (90%) were used for training the model, while the remaining 17 samples (10%) were reserved for model testing. The key variables in this analysis include the laser's velocity, duty cycle, operating frequency, fluency, and peak power, all of which serve as the defining factors. These parameters are associated with the resultant sheet resistance value, providing insight into how laser flux influences LIG resistance. The selected features were varied until a model with an R^2^ value above 0.85 was obtained. To avoid overfitting, a fivefold cross-validation was used. The R^2^ obtained during testing procedures is approximately 0.91 (Supplementary Table S3). In the graph of Fig. 6b, the true values of sheet resistance of the samples (blue points) are compared with the values predicted by the model obtained (yellow points). For ease of interpretation, the data were arranged from lowest to highest according to the real value of sheet resistance, resulting in an ascending progression of the number of observations. This shows that our model is more accurate for values below 2 kΩ/sq. Additionally, scatter plots (predicted response vs. true response) obtained during model training and validation are shown in Fig. 6c,d. These confirm that the bigger dispersion of the predicted values compared to the true values corresponds to the highest resistance.

In addition to R^2^, we have used a statistical test (t-test) to evaluate the performance of the model obtained. This test has been performed on both the training and validation datasets. Its objective is to determine if there is a significant difference between the real values and those predicted by the model. The p-values resulting from the t-test (1 and 0.85) are greater than the certain significance level (α = 0.05), indicating that there is not enough statistical evidence to conclude that there is a significant difference. It suggests that the GPR models obtained are effectively predicting the sheet resistance.

Additionally, the dataset containing all samples previously categorized as conductive was used. The graphs in Supplementary Fig. S4 show that, as in the previous model, there is low accuracy in predicting high sheet resistance values. Supplementary Table S3 shows the R^2^ obtained during the training and testing procedures is approximately 0.77. These results show that the model exhibits superior performance in predicting sheet resistance values below 4 kΩ/sq.

Supplementary Figure S5a fails to provide a distinct illustration of the relationship between the sheet resistance of LIG and laser power. However, when the objective is to attain sheet resistance values below 1 kΩ/sq, it becomes evident that the laser speed must be below 400 mm/s. Additionally, Supplementary Fig. S5c, in the same context, offers valuable insights. This demonstrates a direct relationship: as the laser fluency increases, the resistance decreases, and conversely, as the laser fluence decreases, the resistance levels rise. This observation underscores the pivotal role of laser fluency in shaping the electrical properties of LIG, specifically in terms of its conductivity, making it a crucial factor to consider when aiming for desired resistance characteristics in LIG-based electronic devices.

Supplementary Figure S6 represents the relationship between the sheet resistance of the samples and their morphology. It is observed that most of the samples with PF morphology exhibit high values of sheet resistance (greater than 2 kΩ/sq). On the contrary, in the samples with CN morphology, the majority have values lower than 2 kΩ/sq. In the case of the samples with WF morphology, they are distributed in several ranges of sheet resistance, although the largest number of samples is in the range of intermediate resistance (0.2–2 kΩ/sq).

### Parameter estimation and tuning for desired LIG characteristics

**Figure 7:**
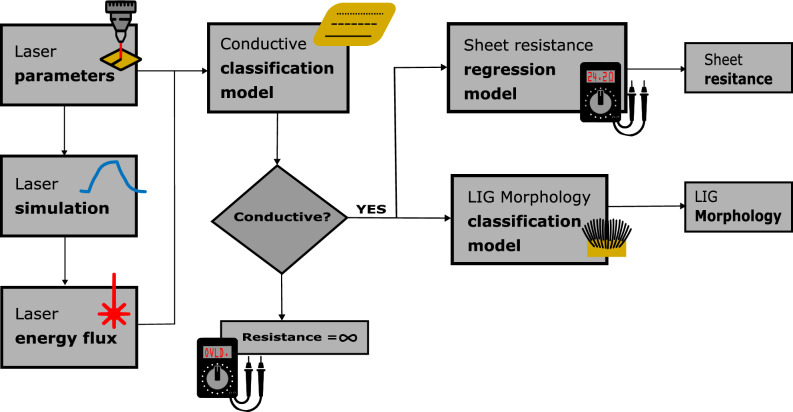
The schematic illustration shows a block diagram of the characteristics of LIG.

Using the models obtained through Simulink simulation, an algorithm has been devised to predict crucial characteristics of LIG. This algorithm provides valuable insight into LIG conductivity, sheet resistance, and morphology, taking into account the myriad configurable and multivariate laser parameters involved in the fabrication process. The algorithm flowchart, as depicted in Fig. 7, outlines its step-by-step operation. Initially, the laser fluency, power density and peak power values are obtained from the laser configurable and fixed parameters through the simulation. These values serve as foundational indicators. Subsequently, the algorithm classifies whether the resulting sample exhibits conductivity or not, acting as a fundamental binary distinction. For samples deemed conductive, the algorithm calculates the sheet resistance, a critical metric in assessing the material's electrical performance. Furthermore, the algorithm also provides insights into the morphology of the conductive LIG sample, enabling researchers and engineers to gain a comprehensive understanding of its structural properties. This predictive algorithm is instrumental in streamlining the LIG fabrication process and allows for informed decision-making in optimizing its properties for specific applications. The codes used for estimation are available in GitHub-link. In the MATLAB script depicted in Supplementary Fig. S6a, the parameters of the laser can be configured; in this way, other researchers can use this algorithm for lasers with different characteristics. Once the parameters are configured, the script is executed, resulting in the estimation of the LIG characteristics (Supplementary Fig. S7b) and a graph simulating the drawing of the LIG line (Supplementary Fig. S7c).

## Conclusions

The successful implementation of LIG in electronic device fabrication remains a complex challenge. However, our research has illuminated a promising path forward by converging advanced simulations and machine learning techniques, all within the framework of digital twins. Through the utilization of simulation-based optimization and the power of digital twins, we have gained invaluable insights into the intricate interplay of laser parameters in LIG production. This understanding not only unravels the complexities of the fabrication process but also sets the stage for fine-tuning LIG properties with precision and control. Crucially, our work introduces a pioneering machine-learning component within the digital twin environment, transforming this knowledge into predictive modeling. These digital twins’ models demonstrate that can predict the conductivity of the LIG network with an accuracy of 96.9%, the morphology of LIG with an accuracy of 88.9%, and estimate the sheet resistance of LIG with an R^2^ value of 0.91. Although the use of a wide range of laser parameters allows generalization of the LIG manufacturing process, a limitation that has been identified within the scope of this research lies in the use of substrates with different characteristics than those of this study. While our study centers on polyimide substrates, the methodology is designed to extend to other materials, inviting future research to explore this versatility. Also, acknowledging the discrepancy between nominal and actual laser parameters, we propose incorporating real-time calibration in future iterations, enhancing the model's predictive accuracy, and bridging the gap between theoretical and empirical outcomes. Additionally, an improvement would be to use a single classification model instead of two, which allows for predicting the successful production of LIG. This model could have four output labels, three for morphology and one for unsuccessful LIG production. Finally, this adaptable digital twin framework demonstrates the potential to inform LIG fabrication with a variety of laser configurations, ensuring broad applicability. This component empowers us to efficiently refine the parameters governing LIG production, enabling us to consistently achieve superior results.

### Supplementary Information

Supplementary Information.

## Data Availability

The datasets generated and analyzed during the study are available at 10.5281/zenodo.10794730.
