# Supplementary material for: Application of digital twins for simulation based tailoring of laser induced graphene
Source: Sci Rep. 2024 May 6;14:10363. doi: 10.1038/s41598-024-61237-6 (PMC11074104; doi:10.1038/s41598-024-61237-6)
Supplement: Supplementary file 1 — Supplementary Information. [file 41598_2024_61237_MOESM1_ESM.pdf]

Supplementary Information

Leveraging Digital Twins for Optimized Fabrication: Simulation-Based Enhancement of Laser-Induced Graphene Writing

José Carlos Santos-Ceballos[1,+], Foad Salehnia[1,*,+] , Alfonso Romero[1], Xavier Vilanova[1]

Universitat Rovira i Virgili, Microsystems Nanotechnologies for Chemical Analysis (MINOS), Tarragona, Spain

Simulation methodology:

In our efforts to provide a tool for tailoring the fabrication of laser-induced graphene (LIG), we have developed an algorithm rooted in our Simulink simulation models that predicts essential LIG characteristics. This algorithm is a critical advancement for the field, as it systematically evaluates and predicts LIG's conductivity, sheet resistance, and morphology based on a range of configurable laser parameters. To convey the algorithm's workflow and its practical applications, we have introduced a flowchart in Figure 1 of the article.

This flowchart begins with the acquisition of foundational laser parameters—fluency, power density, and peak power—from both configurable and fixed settings within the simulation framework. These parameters are crucial as they underpin the LIG's subsequent characteristics. Following this, the algorithm assesses whether the resulting LIG sample is conductive. For conductive samples, it goes a step further to calculate sheet resistance, offering insights critical to the material's electronic utility. It does not stop there; the algorithm also evaluates the morphology of the LIG, providing an integrated view of its structural attributes. The algorithm has been designed to serve not just as an analytical tool but also as a decision-making aid for tailoring LIG properties to specific application needs. The adaptability and depth of our model are encapsulated within the accompanying MATLAB script, which we have made available on GitHub to the research community. This script includes annotations explaining each function and command, ensuring that users can both understand and modify the code as needed for their unique parameters and targets.

This MATLAB script, named simulationLIGFabricatedScript.m on GitHub, is designed to simulate the fabrication process of laser-induced graphene (LIG) and predict its key characteristics. Initially, the script sets both configurable and fixed laser parameters to accurately represent the experimental setup. These include laser power, speed, frequency, and physical attributes like beam size and raising time. It then performs a series of computations to determine the interaction time of the laser with the substrate, the energy delivered per pulse, and the total energy over the drawn line. The heart of the simulation lies within a Simulink model, named laserSim.slx on GitHub, which models the complex dynamics between the laser and the substrate. Post-simulation, the script calculates the energy distribution and fluency, providing insights into the localized effects of the laser on the material properties. Using machine learning models, the script predicts the conductivity and morphology of the LIG samples, such as woolly fibers, cellular networks, or porous formations, and estimates the sheet resistance. These predictions allow for an assessment of the material's electrical performance and structural qualities. The script culminates by visually representing the laser's path and energy deposition, offering an intuitive understanding of the laser writing process.

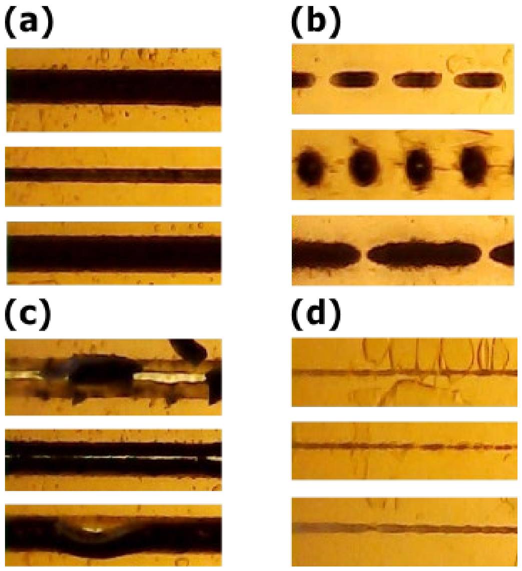

Figure S1. The images of the LIG real samples (a) conductive LIG, (b) not interconnected LIG (classified nonconductive), (c) over-burning Figure S1. The images polymer (classified nonconductive) and (d) melted polymer (classified nonconductive).

Table S1. The table shows the results of the training and validation of the conductivity classification model, for different distributions of the dataset.

Training observations	Validation observations	Training accuracy (%)	Validation accuracy (%)
584 (90%)	60 (10%)	94.7	96.9
519 (80%)	129 (20%)	95.4	91.5
486 (75%)	162 (25%)	93.6	92

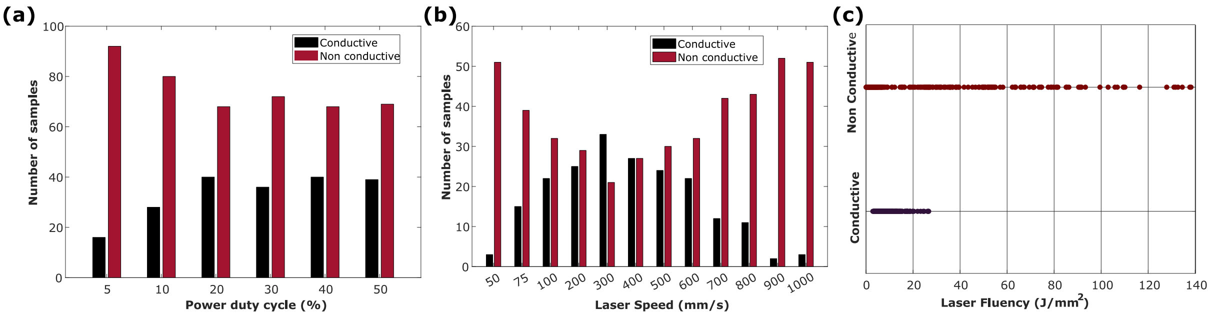

Figure S2. The charts show the relationship between the classification LIG network (conductive, non-conductive) and (a) laser power duty cycle, (b) laser speed, and (c) laser fluency.

Table S2. The table shows the results of the training and validation of the morphology classification model, for different distributions of the dataset.

Training observations	Validation observations	Training accuracy (%)	Validation accuracy (%)
175 (90%)	20 (10%)	86.79	88.9
156 (80%)	39 (20%)	85.9	81.1
146 (75%)	49 (25%)	90.4	83

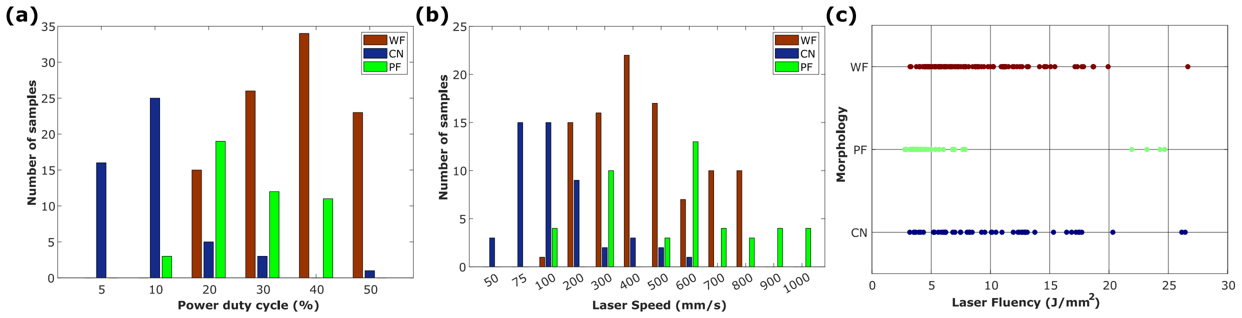

Figure S3. The charts show the relationship between the classification morphology of the LIG and (a) laser power duty cycle, (b) laser speed, and (c) laser fluency.

Table S3. The table shows the results of the training and validation of the estimation LIG sheet resistance model, for different distributions of the dataset.

Training observations	Validation observations	R^2 Training	R^2 Validation
175 (90%)	20 (10%)	0.77	0.77
156 (80%)	39 (20%)	0.66	0.85
146 (75%)	49 (25%)	0.65	0.86
156 (90%)	17 (10%)	0.85	0.91
138 (80%)	35 (20%)	0.85	0.86
130 (75%)	43 (25%)	0.85	0.67

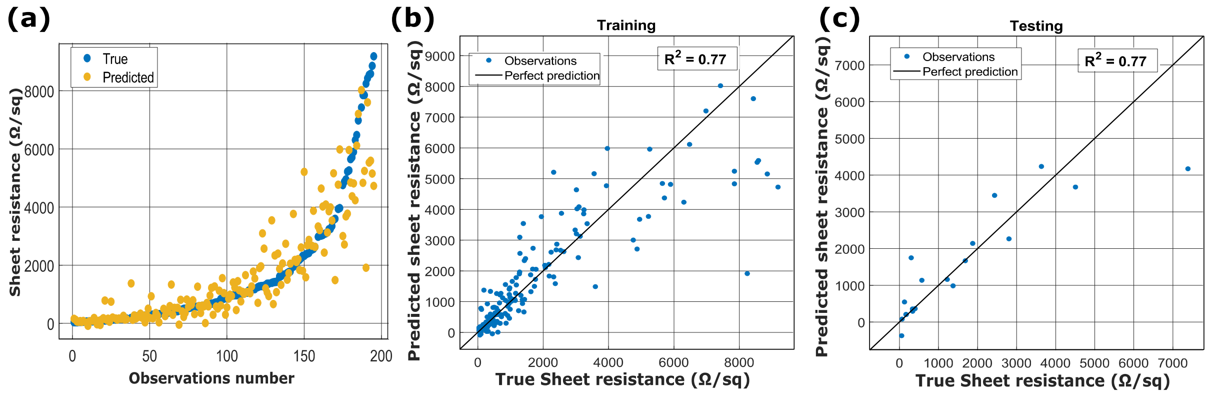

Figure S4. The charts show (a) scatter plots and correlation plots of (b) training and (c) testing procedures for the data set used all classified conductive samples without any resistance cut-off.

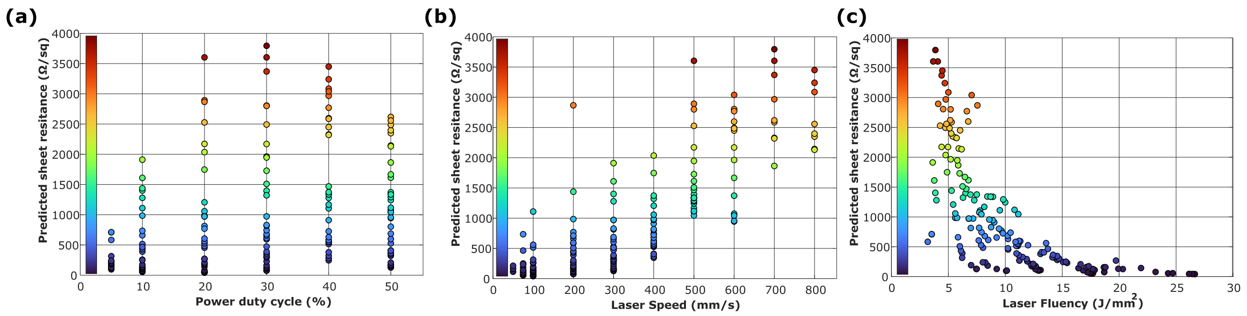

Figure S5. The charts show the relationship between the LIG sheet resistance and (a) laser power duty cycle, (b) laser speed, and (c) laser fluency.

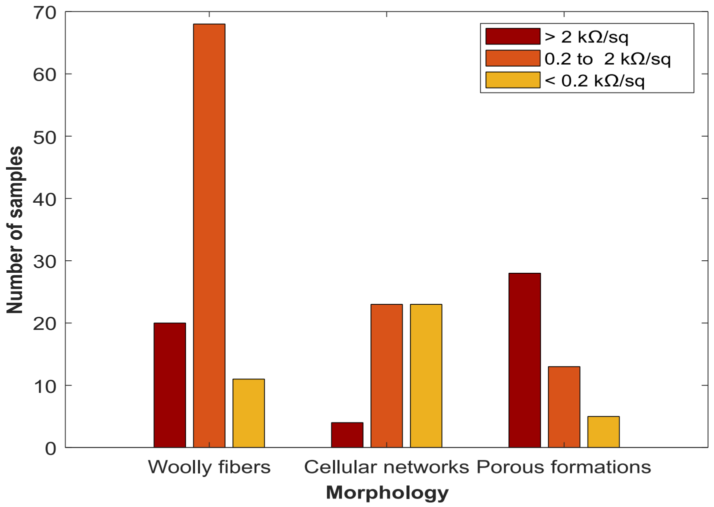

Figure S6. The charts show the relationship between the LIG sheet resistance and their morphology

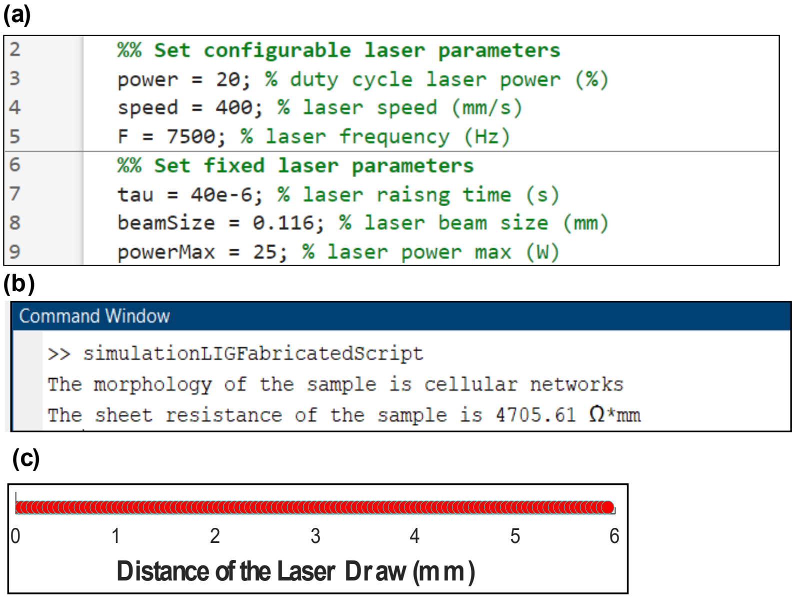

Figure S7. Schematic illustration shows (a) MATLAB script segment used to estimate characteristics LIG, (b) MATLAB command window output when the script is run, and (c) a digital twin graph simulating the drawing of the LIG line.
